# Urinary sodium to guide diuretic therapy in acute heart failure: results of a national survey

**DOI:** 10.1093/eschf/xvag006

**Published:** 2026-01-13

**Authors:** Marta Cobo Marcos, Joan Carles Trullàs, Jose Perez-Silvestre, Òscar Miró, Borja Quiroga, Sonia Mirabet, Gregorio Romero-González

**Affiliations:** Cardiology Department, Hospital Universitario Puerta de Hierro, C/Joaquín Rodrigo 2, 28222, Madrid, Spain; Centro de Investigación Biomédica en Red (CIBER) Cardiovascular, C/ Monforte de Lemos 3-5, Pabellón 11, 28029 Madrid, Spain; Internal Medicine Department, Hospital dOlot i comarcal de la Garrotxa, Girona, Spain; Laboratori de Reparació i Regeneració Tissular (TR2Lab), Facultat de Medicina, Universitat de Vic—Universitat Central de Catalunya, Barcelona, Spain; Internal Medicine Department, Consorcio Hospital General Universitario de Valencia, Valencia, España; Department of Emergency, Hospital Clínic, ‘Processes and Pathologies, Emergencies Research Group’ IDIBAPS, University of Barcelona, Barcelona, Spain; IIS-La Princesa, Nephrology Department, Hospital Universitario de la Princesa, RICORS-renal 2040, Madrid, Spain; Centro de Investigación Biomédica en Red (CIBER) Cardiovascular, C/ Monforte de Lemos 3-5, Pabellón 11, 28029 Madrid, Spain; Cardiology Department, Hospital Santa Creu i Sant Pau, Universitat Autonoma de Barcelona, Barcelona, Spain; Nephrology Department, Medicine Department, University Hospital Germans Trias i Pujol, (HGiTP) & REMAR-IGTP Group, Germans Trias i Pujol Research Institute (IGTP), Universitat Autònoma de Barcelona, Can Ruti Campus, Badalona, Spain; International Renal Research Institute of Vicenza, Vicenza, Italy

**Keywords:** Urinary sodium, uNa^+^, Natriuresis, Acute heart failure

## Abstract

**Background and Aims:**

Urinary sodium (uNa^+^) is a promising tool to guide diuretic therapy in acute heart failure (AHF), yet its real-world adoption is uncertain. We aimed to evaluate current practices, perceived utility, and barriers to uNa⁺-guided management among physicians managing AHF in Spain.

**Methods:**

We conducted a nationwide cross-sectional electronic survey in June 2025 of clinicians from cardiology, internal medicine, emergency medicine, and nephrology. The questionnaire assessed the frequency and timing of uNa^+^ monitoring in inpatient and outpatient settings, thresholds for poor response, therapeutic adjustments, and perceived benefits and barriers.

**Results:**

A total of 413 physicians responded: 138 (33%) from cardiology, 159 (39%) from internal medicine, 78 (19%) from emergency medicine, and 38 (9%) from nephrology. Overall, 70% reported measuring uNa^+^ during AHF hospitalization, but only 18% did so routinely. Outpatient application remained infrequent (32%). Nephrologists showed the highest usage in both inpatient (90%) and outpatient (61%) settings. The most frequent cut-off to define diuretic resistance was <50 mmol/l. Therapeutic strategies differed by specialty: loop diuretic escalation was more frequent in cardiology and emergency medicine, while thiazides were preferred in internal medicine and nephrology. Most participants (78%) considered uNa⁺ monitoring clinically useful. Major barriers included lack of standardized protocols, limited training, therapeutic inertia, and logistical constraints.

**Conclusions:**

While uNa⁺ monitoring is valued by clinicians, its use in AHF in Spain remains inconsistent across specialties. Addressing implementation barriers through education, protocols, and decision-support tools is critical to a broader adoption of uNa⁺ guided therapy in routine care.

## Background

Congestion is the predominant clinical manifestation of acute heart failure (AHF) and a leading cause of hospitalization.^1^ Diuretics remain the cornerstone of therapy to increase sodium excretion and achieve euvolemia.^2^ However, many patients exhibit diuretic resistance, which is associated with worse outcomes.^3^ Urinary sodium (uNa^+^) has emerged as a key objective marker of diuretic response, with low post-diuretic uNa^+^ levels associated with worsening kidney function, persistent congestion, and adverse events.^4–10^ Consistent with this, recent randomized and observational studies using uNa^+^-guided algorithms have shown improved natriuresis/diuretic efficiency and faster decongestion with this strategy.^11,12^ Accordingly, expert consensus and European guidelines recommend using uNa^+^ to guide diuretic therapy in AHF.^2,13,14^ Nonetheless, its implementation in clinical practice and variations across specialties remain unclear.

## Aims

To address the lack of data on real-world practices, we conducted a nationwide survey within the framework of an initiative led by national scientific societies to evaluate the use of uNa^+^-guided diuretic therapy in AHF. The objective was to examine and compare clinical practices, perceptions, and barriers to implementation across key specialties involved in AHF management, including cardiology, internal medicine, emergency medicine, and nephrology.

## Methods

A cross-sectional electronic survey was conducted in June 2025. The questionnaire included multiple-choice and Likert-style items on the use of uNa^+^ across several domains: use of uNa^+^ in inpatient and outpatient settings, timing of measurement, thresholds used to define inadequate natriuretic response, therapeutic actions in response to low uNa^+^ values, and perceptions regarding its clinical utility and implementation barriers.

The coordinators of the national Heart Failure or Cardiorenal working groups of each specialty determined the distribution strategy. For internal medicine and cardiology, the questionnaire was distributed to all members of each group’s mailing list (2122 internists and 2366 cardiologists). For emergency medicine, a survey was sent to the emergency physicians who act as local principal investigators at 60 Spanish hospitals, who then shared it with the rest of the staff. Roughly, we estimate that the survey reached about 1000 emergency physicians. Finally, in the case of nephrology, the survey was sent to the heads of departments in 178 hospitals (around 70% of Spanish public hospitals), who delegated it to the physician primarily responsible for heart failure/cardiorenal syndrome management.

Responses were collected anonymously, and participation was voluntary. Physicians attending patients with AHF and practising in various levels of care (including heart failure units, hospital wards, and emergency departments) were eligible to participate. Collected data were summarized using descriptive statistics. Categorical variables were expressed as frequencies and percentages. Differences in responses across specialties were evaluated using χ^2^ tests, with statistical significance defined as *P* < .05. Statistical analyses were performed using Stata 15.1 (Stata Statistical Software, Release 15, 2017; StataCorp LP, USA).

## Results (*Table 1*)

A total of 413 physicians from 165 hospitals in Spain completed the survey: 138 (33%) from cardiology, 159 (39%) from internal medicine, 78 (19%) from emergency medicine, and 38 (9%) from nephrology.

**Table 1:** Urinary sodium-guide diuretic therapy survey main results

Do you use urinary sodium measurement as a guide for diuretic treatment in patients with acute heart failure?						
Specialty	*N*	Always/almost always	In more than 50%	In less than 50%	Never/almost never	*P*-value
Total	413	(74, 18%)	(77, 19%)	(137, 33%)	(125, 30%)	<.001
Cardiology	138	(14, 10%)	(29, 21%)	(54, 39%)	(41, 30%)	
Int. Med.	159	(27, 17%)	(33, 21%)	(52, 33%)	(47, 29%)	
Nephrology	38	(12, 32%)	(8, 21%)	(14, 37%)	(4, 10%)	
Emerg. Med.	78	(21, 27%)	(7, 9%)	(17, 22%)	(33, 42%)	

Do you use this strategy in the outpatient setting?						
Specialty	*N*	Always/almost always	In more than 50%	In less than 50%	Never/almost never	*P*-value
Total	397	(24, 6%)	(28, 7%)	(76, 19%)	(269, 68%)	<.001
Cardiology	136	(6, 4%)	(9, 7%)	(26, 19%)	(95, 70%)	
Int. Med.	156	(9, 6%)	(11, 7%)	(35, 22%)	(101, 65%)	
Nephrology	38	(6, 16%)	(6, 16%)	(11, 29%)	(15, 39%)	
Emerg. Med.	67	(3, 4%)	(2, 3%)	(4, 6%)	(58, 87%)	

What urinary sodium threshold do you use to define diuretic resistance?						
Specialty	*N*	<50 mmol/l	<70 mmol/l	<100 mmol/l	Do not know	*P*-value
Total	403	195 (48%)	141 (35%)	14 (4%)	53 (13%)	<.001
Cardiology	138	80 (58%)	48 (35%)	2 (1%)	8 (6%)	
Int. Med.	155	69 (45%)	66 (41%)	4 (2%)	22 (12%)	
Nephrology	37	22 (59%)	9 (24%)	4 (11%)	2 (5%)	
Emerg. Med.	73	24 (33%)	20 (27%)	5 (7%)	24 (33%)	

What is your most frequent response to a low urinary sodium value?						
Specialty	*N*	Increase loop diuretic	Add thiazide	Add acetazolamide	Make no change	*P*-value
Total	396	220 (56%)	145 (37%)	11 (3%)	20 (5%)	<.001
Cardiology	135	84 (62%)	47 (35%)	3 (2%)	1 (1%)	
Int. Med.	150	74 (49%)	65 (43%)	3(2%)	8 (5%)	
Nephrology	38	17 (45%)	18 (47%)	2 (5%)	1 (3%)	
Emerg. Med.	73	45 (62%)	15 (20%)	3 (4%)	10 (14%)	

Int. Med, internal medicine; Emerg. Med, emergency medicine

### Use of urinary sodium in hospitalized patients with acute heart failure

Overall, 288 (70%) respondents reported utilizing uNa⁺ measurement to guide intravenous diuretic therapy during hospitalization for AHF, 74 (18%) reported using it ‘always or almost always,’ 77 (19%) applied it ‘in more than 50% of cases’, and 137 (33%) in less than 50% of cases. The remaining 125 physicians (30%) stated they never adopted this approach.

### Use of urinary sodium in the outpatient setting

In contrast, utilization of uNa⁺ in the outpatient or follow-up setting was considerably lower. Only 128 (32%) of respondents indicated any application of uNa⁺ monitoring, but just 24 of them (6%) reported using it routinely.

### Variation in practice across specialties

We observed significant differences in inpatient implementation of uNa⁺-guided therapy across specialties (*P* < .001). At least occasional utilization was noted among 70% of cardiologists and internists. This proportion increased to 90% among nephrologists, who were also the most likely to apply it routinely (32% ‘always or almost always’). In contrast, only 58% of emergency medicine physicians indicated any use of uNa⁺. Similar differences between specialties were also observed in the outpatient setting, where any application ranged from 13% in emergency medicine to 61% among nephrologists.

Beyond specialty, we also assessed variation in uNa⁺ strategies based on the respondents’ primary work setting, including heart failure or cardiorenal units, hospital wards, outpatient clinics, or combined roles. Among these non-emergency care settings, we did not find statistically significant differences in the inpatient application of uNa^+^ to guide diuretic therapy (*P* = .341), suggesting a relatively consistent level of adoption across clinical environments.

### Thresholds used to define diuretic resistance

The most commonly selected threshold used to define poor diuretic response was <50 mmol/l (48%), followed by <70 mmol/l (35%). Uncertainty regarding the appropriate threshold was reported by 13% of respondents, and this was particularly pronounced among emergency physicians (33%).

### Therapeutic strategies in response to low urinary sodium

When managing low uNa⁺ levels, 56% of physicians stated they would increase the dose of the loop diuretic, while 37% would add a thiazide-type diuretic. Far fewer indicated they would add acetazolamide (3%) or make no change to treatment (5%). These strategies significantly differed by specialty (*P* < .001): loop diuretic escalation was more common among cardiologists and emergency physicians, whereas thiazide use was more frequent among internists and nephrologists.

### Perceived clinical utility

Most respondents (78%) considered uNa⁺ monitoring to be a valuable tool for optimizing diuretic therapy. A smaller proportion (18%) were unsure, and only 4% believed it was not helpful. Key perceived benefits included enhanced natriuresis or diuresis (70%), faster decongestion (67%), and the identification of low urinary sodium as a marker of poor prognosis or diuretic resistance (45%). Additionally, 35% believed that the use of uNa⁺ could potentially reduce rehospitalizations.

### Barriers to implementation

We identified several barriers to broader adoption of uNa^+^ strategy. The most commonly reported issues were the absence of standardized protocols (59%), a lack of training or knowledge (52%), therapeutic inertia (51%), and logistical or workflow-related limitations (48%). These barriers also varied by specialty: emergency physicians were more likely to cite gaps in training and workflow difficulties, whereas cardiologists and internists more often emphasized the lack of clear protocols. Notably, nearly half of respondents overall reported a perceived low clinical priority, which was particularly frequent among nephrologists (58%) and emergency physicians (47%).

## Discussion

This national survey reveals marked variability in the adoption of uNa⁺ monitoring to guide diuretic therapy in AHF across medical specialties in Spain. While two-thirds of cardiologists and internists and the majority of nephrologists reported at least occasional use, adoption was lower among emergency physicians, likely reflecting differences in familiarity, workflow constraints, and training.

Encouragingly, respondents across specialties broadly acknowledged the value of natriuresis-guided diuresis. Most specialists identified a post-diuretic uNa⁺ threshold of 50–70 mmol/l as indicative of insufficient diuretic response, consistent with prior observations.^2,13,14^ Likewise, there was agreement that low uNa⁺ warrants intensification of therapy. Differences in preferred interventions likely relate to training and practice patterns: cardiologists and emergency physicians tended to maximize loop diuretic dosing first, whereas internal medicine and nephrology specialists more readily added a thiazide. The infrequent selection of acetazolamide suggests limited awareness of its emerging role despite growing supporting evidence.^15^

Recent prospective studies have provided clinical validation for uNa⁺-guided diuretic strategies. The ENACT-HF trial,^11^ a pragmatic multicentre study, demonstrated that a standardized uNa⁺-guided protocol significantly increased natriuresis by 64% within 24 h compared to standard care, and was associated with greater overall diuresis and a shorter hospital stay, without increasing adverse renal or haemodynamic events. Similarly, the PUSH-AHF trial,^12^ a randomized single-centre study, confirmed that uNa⁺-guided therapy improved early decongestion parameters; however, it did not significantly impact 180-day heart failure rehospitalizations. These findings support the physiological basis and current guideline recommendations for using uNa⁺ to guide diuretic adjustment in AHF, showing its potential to improve short-term management. This growing body of evidence has likely contributed to the generally positive attitudes towards uNa⁺ monitoring observed across specialties in our survey. However, the identified barriers help to explain why uNa⁺ guidance is not yet standard practice. Knowledge gaps and the absence of standardized protocols are remediable obstacles. Targeted education and structured integration of uNa⁺ monitoring into heart failure care pathways could reduce inter-specialty variability and promote more consistent use.

In this context, a 2025 expert consensus from the European Society of Cardiology has taken a pivotal step forward by proposing practical algorithms for diuretic titration based on uNa⁺ thresholds, and advocating for point-of-care testing to support real-time clinical decisions.^14^ Such initiatives underscore the growing recognition of this strategy’s clinical value and provide a framework to support its broader implementation. Nonetheless, many clinicians still cited therapeutic inertia and perceived low priority for uNa⁺ monitoring, highlighting the need to continue generating robust evidence and to actively reposition natriuresis-guided therapy as a central element in AHF management.

An important limitation of this study is the potential for selection bias. Given the voluntary, survey-based design, respondents were likely those more actively engaged in AHF management and more familiar with uNa⁺-guided therapy, which may overestimate its real-world use. A second source of bias stems from the uneven distribution strategy across specialties. Together with the modest overall response rate (<10%), this likely contributed to underrepresentation of nephrologists and reinforces that the findings primarily reflect the views of more engaged clinicians rather than the broader practising population.

## Conclusions

In conclusion, the use of uNa⁺ to guide diuretic therapy in AHF in Spain shows considerable variation across medical specialties. While most practitioners recognize its potential to enhance decongestion, its routine implementation remains uneven. Addressing the educational gaps and developing standardized protocols, along with ongoing evidence generation, will be key to expanding uNa⁺-guided diuretic therapy in clinical practice.
